# Disseminated Intra-abdominal and Pelvic Abscesses Caused by Streptococcus pneumoniae and Staphylococcus epidermidis in a Previously Healthy Adolescent

**DOI:** 10.7759/cureus.107392

**Published:** 2026-04-20

**Authors:** Astha Poudel, Sharmila Lamichhane, Sydney E Shaffer, Haris Javed

**Affiliations:** 1 Pediatrics, Mercy Health - St. Vincent Pediatric Residency, Toledo, USA; 2 General Practice, Bharatpur Hospital, Chitwan, NPL; 3 Pediatrics, Ohio University Heritage College of Osteopathic Medicine, Athens, USA; 4 Pediatrics, Nationwide Children’s Hospital, Toledo, USA

**Keywords:** immunocompetent individuals, inflammatory bowel, intraabdominal abscess, prolonged antibiotics, staphylococcus epidermidis, streptococcus pneumoniae

## Abstract

A 14-year-old healthy boy, initially treated for presumed muscle strain, developed progressive left leg pain, a groin mass, and a 30-pound weight loss over six weeks. Labs showed leukocytosis, anemia, thrombocytosis, and elevated inflammatory markers. CT revealed multiloculated pelvic and iliopsoas abscesses with sacroiliac septic arthritis, osteomyelitis, and epidural extension. He underwent a laminectomy, surgical and CT-guided drainage, and prolonged targeted antibiotics. Cultures grew *Streptococcus pneumoniae* and *Staphylococcus epidermidis*. Extensive workup for immunodeficiency, Crohn's disease, and malignancy was negative. Atypical invasive infection should be considered even in the absence of traditional risk factors. Early imaging, source control, and prolonged antimicrobial therapy are critical in managing complex deep-seated abscesses.

## Introduction

*Streptococcus pneumoniae* is an uncommon cause of deep-seated intra-abdominal abscesses, particularly in immunocompetent male teenagers [1,2]. The presence of multiple pelvic and intra-abdominal abscesses with associated osteomyelitis and septic arthritis in an otherwise healthy adolescent presents a significant diagnostic challenge and raises concern for occult infection, immunodeficiency, malignancy, or inflammatory disease [3,4].

## Case presentation

**Figure 1:**
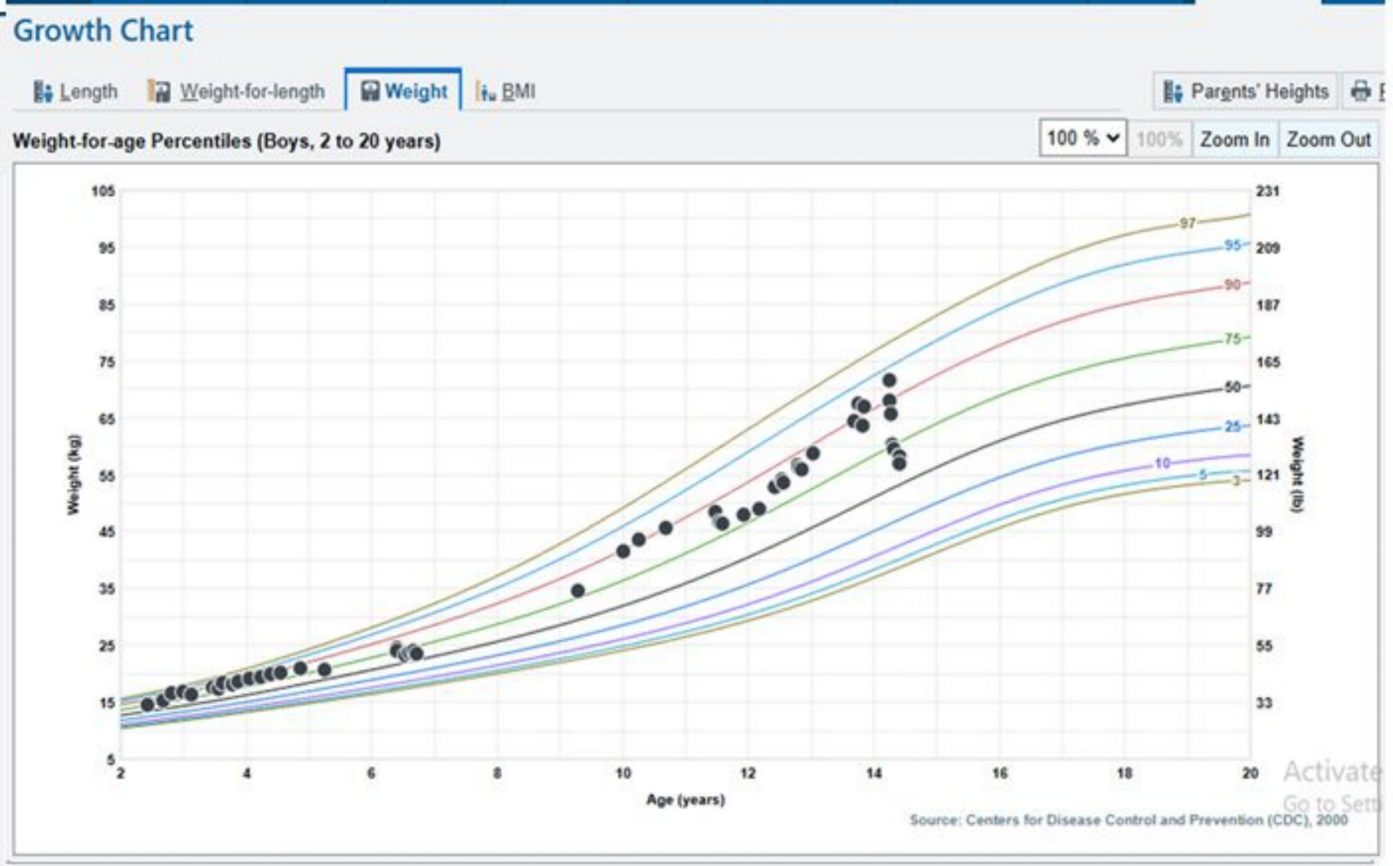
Significant weight loss, crossing two percentiles over a short period of time.

**Figure 2:**
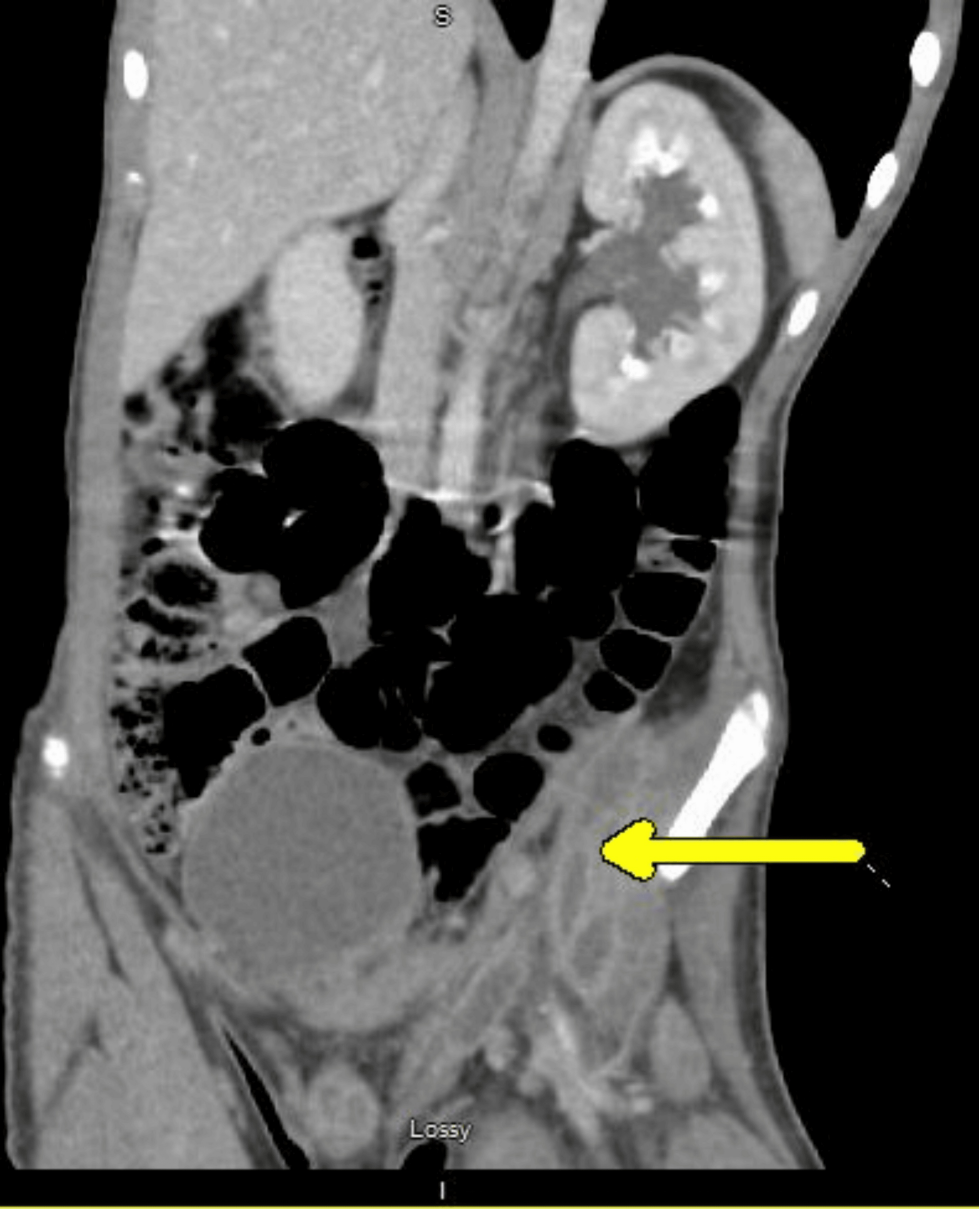
Left perivesical and left perirectal collection.

**Figure 3:**
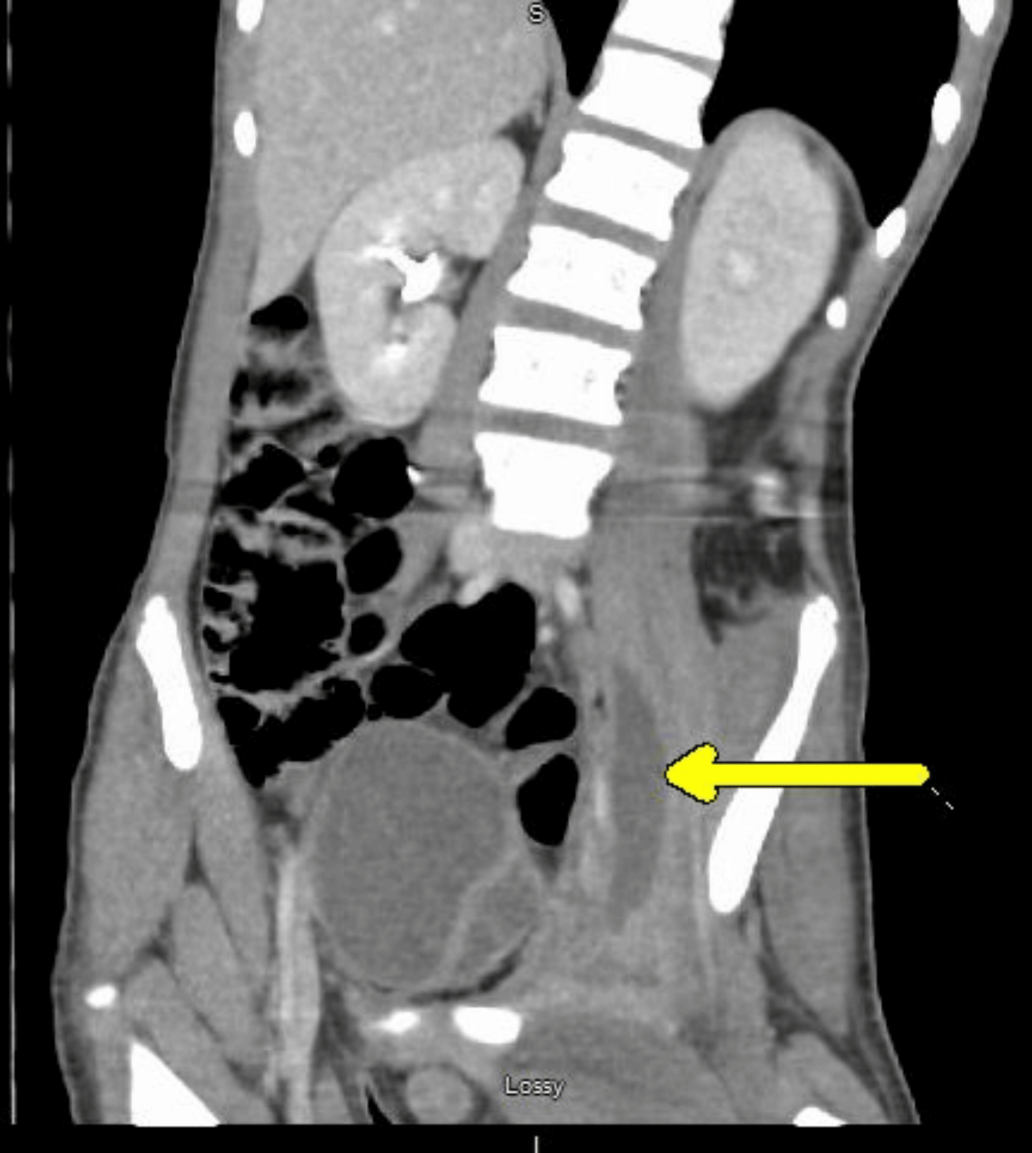
Abscess in the left iliopsoas region, likely with extension into the inflammatory process in the left inguinal/groin region.

A 14-year-old boy received care for a presumed muscle strain after experiencing left lower extremity pain following a sports camp. He was treated with over-the-counter pain relievers and physical therapy. Hip X-rays revealed no acute osseous abnormality. Over the duration of a month, his pain worsened, and he could not tolerate physical therapy. A magnetic resonance imaging (MRI) scan was ordered, but he could not tolerate lying flat to complete the study. Approximately six weeks after his initial symptoms, he presented to the emergency department with a painful left groin mass. He also reported an unintentional weight loss of 30 pounds (Figure 1). Laboratory studies showed leukocytosis (white blood cells = 22.5 x 109/L), thrombocytosis (797 x 109/L), anemia (hemoglobin = 7.8 g/dL), and elevated inflammatory markers (erythrocyte sedimentation rate = >130 mm/hr; C-reactive protein = 343 mg/L) (Table 1). Computed tomography (CT) of the abdomen and pelvis demonstrated multiple multiloculated abscesses involving the left iliacus and iliopsoas muscles, perivesical, perirectal, presacral, and posterior sacral regions, with gas formation; left sacroiliac septic arthritis; osteomyelitis of the left iliac bone; hydronephrosis; and concern for epidural extension (Figures 2, 3). Blood, urine, and left groin wound cultures were obtained. The patient had no significant history of immunodeficiency, IV drug use, recurrent infections, or immunosuppressive medication use. He was up to date on vaccines. Family history was also noncontributory.

**Table 1:** Significant lab values with normal reference range.

Lab value	Result	Reference range
WBC	22.5 k/uL	4.5-13.5 k/uL
Hemoglobin	7.8 g/dL	13-15 g/dL
Platelet	797 k/uL	138-453 k/uL
Neutrophil %	81%	34-64%
Absolute neutrophil	18.06 k/uL	1.50-8.00 k/uL
Lactic acid	2.4 mmol/L	0.5-2.2 mmol/L
Procalcitonin	0.3 ng/mL	<0.5 ng/mL (Low likelihood of sepsis)
Sedimentation rate	>130 mm/hr	0-15 mm/hr
C-reactive protein	343.0 mg/L	0-5 mg/L
Blood culture	No growth	No growth
Stool calprotectin	1570 µg/g	<49 µg/g
IgG	2380 mg/dL	700-1600 mg/dL
IgA	370 mg/dL	47-249 mg/dL
IgM	107 mg/dL	15-188 mg/dL

The patient underwent epidural abscess debridement and L5-S1 laminectomy. Two days later, he had CT-guided drainage of the left pelvic abscess. The lumbar subcutaneous abscess culture grew *Staphylococcus epidermidis*, and the sacral abscess culture grew *Streptococcus pneumoniae*. Further tests like transthoracic echocardiography (TTE) and magnetic resonance enterography (MRE) were nondiagnostic, though the patient had raised fecal calprotectin (1570 ug/g). Blood culture did not show any growth. The patient required blood transfusions; therefore, limiting the ability for an immunological work-up. The patient was treated with broad-spectrum intravenous antibiotics (vancomycin, ceftriaxone, and metronidazole) for two weeks, followed by step-down oral therapy (sulfamethoxazole and trimethoprim ) for four more weeks, with antibiotic selection based on culture and sensitivity, and had clinical improvement. After discharge, the patient underwent endoscopy and colonoscopy, which were negative.

## Discussion

This case illustrates a rare presentation of disseminated intra-abdominal, pelvic, and epidural abscesses caused by *Streptococcus pneumoniae* in an otherwise healthy adolescent [5,6]. The differential diagnosis included secondary bacterial infection, Crohn's disease, immunodeficiency, and hematologic malignancy, all of which were systematically ruled out through history, laboratory testing, imaging, and endoscopic evaluation [7,8]. The patient’s nonspecific initial symptoms, comprising progressive musculoskeletal pain and weight loss, highlight the diagnostic challenge of deep-seated abscesses in children. The most common causes for intra-abdominal abscesses in the pediatric population are polymicrobial, with *Escherichia coli* (aerobe) and *Bacteroides fragilis* (anaerobe) being the most common. While *S. pneumoniae* typically causes respiratory infections and *S. epidermidis* is an opportunistic skin commensal, their involvement here demonstrates that atypical invasive infections can occur even in immunocompetent hosts [9]. *Streptococcus pneumoniae* has been associated with intrabdominal abscess in females through ascending genital tract infection. Also, pneumococcal peritonitis is classically associated with underlying conditions like cirrhosis, nephrotic syndrome, and immunodeficiency. Gastrointestinal translocation, though rare, is possible secondary to chronic gastric acid suppression, recent endoscopy, underlying inflammatory bowel disease, and recent intra-abdominal surgery, which were all absent in this case. Early cross-sectional imaging, prompt surgical or percutaneous drainage, and prolonged targeted antimicrobial therapy were critical to successful management [10]. Clinicians should maintain a high index of suspicion for complex intra-abdominal and pelvic abscesses in children presenting with progressive pain, systemic symptoms, or unexplained weight loss, even in the absence of traditional risk factors.

## Conclusions

Severe intra-abdominal and pelvic abscesses can occur in otherwise healthy children without typical risk factors. Prompt imaging, effective source control, and targeted antimicrobial therapy are essential for successful outcomes. Clinicians should remain vigilant for deep-seated infections caused by atypical pathogens, even when presenting with nonspecific symptoms such as musculoskeletal pain or significant weight loss.
